# Hidden in Plain Sight: Discovering Giant Cell Aortopathy During Surgical Mitral Valve Repair

**DOI:** 10.1016/j.jaccas.2022.03.014

**Published:** 2022-05-04

**Authors:** Najah A. Khan, Daniel Li, Emily Newstrom, Roberto Barrios, Mohammed Attar

**Affiliations:** a Department of Internal Medicine, Houston Methodist Hospital, Houston, Texas, USA; b Texas A&M College of Medicine, Bryan, Texas, USA; c Department of Pathology, Houston Methodist Hospital, Houston, Texas, USA; d DeBakey Heart and Vascular Institute, Houston Methodist Hospital, Houston, Texas, USA

**Keywords:** aortic aneurysm, aortopathy, giant cell arteritis, inflammatory marker, mitral valve prolapse, positron emission tomography, thoracic ascending aorta, vasculitis, CRP, C-reactive protein, ESR, erythrocyte sedimentation rate, GCA, giant cell arteritis, MR, mitral valve regurgitation, MRI, magnetic resonance imaging, PET-FDG, positron emission tomography–fluorodeoxyglucose

## Abstract

Giant cell arteritis (GCA) is an inflammatory cranial and/or extracranial vasculitis. Although cranial GCA is widely recognized, extracranial GCA is underdiagnosed because of its nonspecific and atypical presentations. We report a case of asymptomatic extracranial GCA with ascending thoracic aortopathy discovered incidentally during surgical mitral valve repair. (**Level of Difficulty: Intermediate.**)

## Central Illustration

**Figure:**
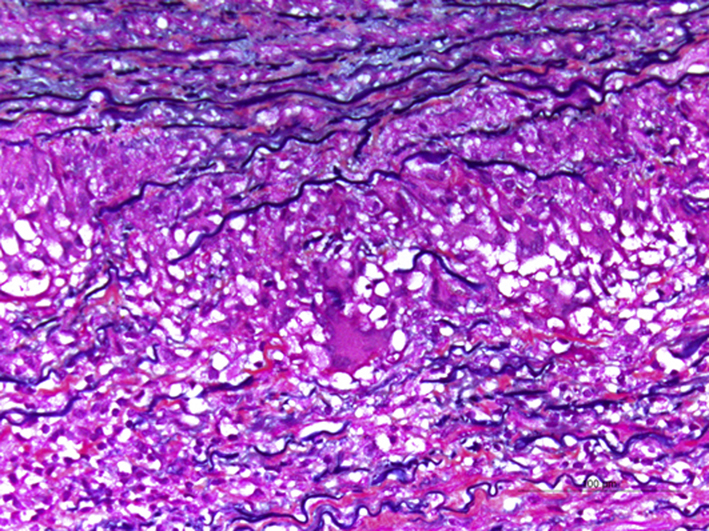

## History of Presentation

A 69-year-old Hispanic man with a medical history of mitral regurgitation (MR) secondary to new-onset mitral valve prolapse, paroxysmal atrial fibrillation, chronic kidney disease stage G3, hyperlipidemia, and hypertension presented to the hospital with a 1-week history of persistent dyspnea at rest without chest pain, orthopnea, peripheral edema, or back pain. He also did not describe headaches, fevers, weight loss, or vision changes. On arrival, his blood pressure was 130/80 mm Hg, heart rate was 90 beats/min, oxygen saturation on room air was 95%, with respirations at 18 breaths/min. The results of physical examination were remarkable for a 3/6 diastolic murmur at the apex, consistent with MR.

**Learning Objectives**

• To recognize variable presentations and diagnostic challenges of extracranial giant cell arteritis including asymptomatic or nonspecific presentations and discordant inflammatory markers.
• To understand the utility and efficacy of different imaging modalities in evaluating extracranial giant cell arteritis, especially advanced imaging (computed tomography, magnetic resonance imaging angiography, and/or positron emission tomography–fluorodeoxyglucose).

## Medical History

He did not describe a personal or family history of myocardial infarction, heart failure, stroke, coagulopathy, or inflammatory conditions, including rheumatic heart disease. He had recently received a diagnosis of mitral valve prolapse with moderate MR at an outside hospital.

## Differential Diagnosis

The cardiac differential diagnosis for his shortness of breath included acute primary versus secondary MR, pulmonary hypertension, new-onset heart failure, and dysrhythmia.

## Investigations

Laboratory results showed normal troponin, B-type natriuretic peptide, and D-dimer levels, borderline erythrocyte sedimentation rate (ESR) of 33 mm/h and C-reactive protein (CRP) of 0.71 mg/dL. Electrocardiography showed normal sinus rhythm. Chest x-ray showed cardiomegaly without mediastinal widening, infiltrates, or opacities. Initial imaging included a noncontrast 3D echocardiogram with a 65% left ventricular ejection fraction, severe MR, and moderate mitral valve leaflet thickening with myxomatous degeneration without vegetations. There was no aortic regurgitation or root dilation and insufficient tricuspid regurgitation jet to measure pulmonary arterial systolic pressures. Left heart catheterization showed no obstructive coronary artery disease. Carotid ultrasound showed intimal carotid thickening bilaterally with <50% stenosis. Noncontrast chest computed tomography revealed a 4.9-cm thoracic ascending aortic dilation.

## Management

**Figure 1:**
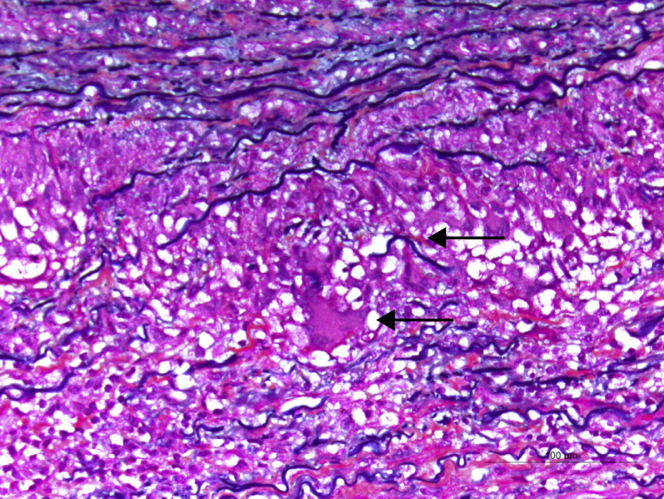
Microscopic View of Ascending Aortic Specimen There is medial necrosis with inflammatory cells including multinucleated giant cells **(bottom arrow)** with destruction of the elastic fibers **(top arrow)** demonstrated by Movat and Veroeff-Van Gieson stains.

The patient underwent surgical mitral valve repair. Incidentally, a 5.5-cm thoracic aortic ascending aneurysm without dissection was discovered intraoperatively and required repair. The postoperative course was complicated by a transient junctional block and moderate pericardial effusion that resolved with medical management. An aneurysmal surgical biopsy specimen showed giant cell arteritis (GCA) (Figure 1). The patient underwent a positron-emission tomography-fluorodeoxyglucose (PET-FDG) study showing no FDG uptake in other large arteries, excluding other vessel involvement.

## Discussion

### Extracranial GCA: clinical presentation and complications

Giant cell arteritis may present with cranial and/or extracranial vessel involvement. Cranial GCA involves temporal or carotid arteries, commonly contributing to temporal headaches, vision loss, and/or jaw claudication and is associated with elevated inflammatory markers. Extracranial GCA involves the aortic arch, axillary, subclavian, femoral, and distal arteries.^1^ It may present asymptomatically or with nonspecific symptoms such as fever and shortness of breath in 1/3 to 2/3 of patients with biopsy-proven GCA.^2^^,^^3^ Significant symptoms include a new murmur or severe localized pain (depending on vessel involvement).^1^ Complications include distal arterial claudication or stenosis and aortic aneurysm, dissection, or insufficiency.^1^ Risk factors for aneurysmal progression include hypertension and atherosclerosis.^4^ In a study involving 974 GCA patients, 45% had aortopathy, with 69% having aortic aneurysm or dilation and 6% experiencing dissection; a 1.5 mm/y average aneurysmal growth rate was calculated in GCA patients, higher than degenerative aortic diseases.^5^ Our patient was considered asymptomatic because his symptoms were likely caused by severe MR in the absence of symptoms suggesting aortic involvement (new murmur or severe localized pain), making the diagnosis of extracranial GCA difficult.

### Diagnostic challenges: epidemiology and laboratory studies

Because of the underrecognition of extracranial GCA, there are limited data evaluating its incidence and prevalence; the estimated prevalence is 3% to 15% up to 92% based on smaller studies.^1^ Moreover, no study has evaluated whether elevated inflammatory markers (ESR and CRP) have the same sensitivity in diagnosing extracranial GCA as cranial GCA^1^ However, extracranial GCA may have lower CRP levels^2^ or may have discordant markers in comparison with cranial GCA (our patient’s ESR was mildly elevated, but CRP was normal),^6^ complicating diagnosis and necessitating further evaluation with imaging.

### Diagnostic challenges: imaging and biopsy

Although the gold standard for diagnosing cranial GCA is temporal artery biopsy, its sensitivity is below 58% in diagnosing extracranial GCA.^1^ Ultrasonography is anatomically limited in evaluating distal aortic disease because of the chest cavity but can evaluate aortic valvular abnormalities. Although 2D echocardiography led to the detection of severe MR, the study was not ordered to examine and measure thoracic aortic measurements; thus, the aorta was not visualized above the sinotubular junction. According to the 2010 American College of Cardiology thoracic aortic disease guidelines, initial evaluation for large vessel vasculitis involves CT or magnetic resonance imaging (MRI).^7^ Noncontrast CT lacks temporal and spatial resolution, rendering aortic measurements inaccurate because of aortic motion and poor aortic spatial mapping compared with CT angiography, which can better detect aneurysm and stenosis;^9^ this can explain the discrepancy in aortic dimensions between the noncontrast CT and the results of intraoperative evaluation in our patient. The European League Against Rheumatism introduced diagnostic guidelines in 2018 emphasizing the use of CT, MRI, or PET-FDG in aiding the diagnosis of extracranial GCA. MRI angiography has high sensitivity (81%) and specificity (97%),^8^ although PET-FDG has a higher sensitivity (83%) and specificity (67% to 100%) in detecting thoracic aortic disease^8^^,^^9^ and is also used to evaluate extra-aortic involvement (as used in our case).

**Figure 2:**
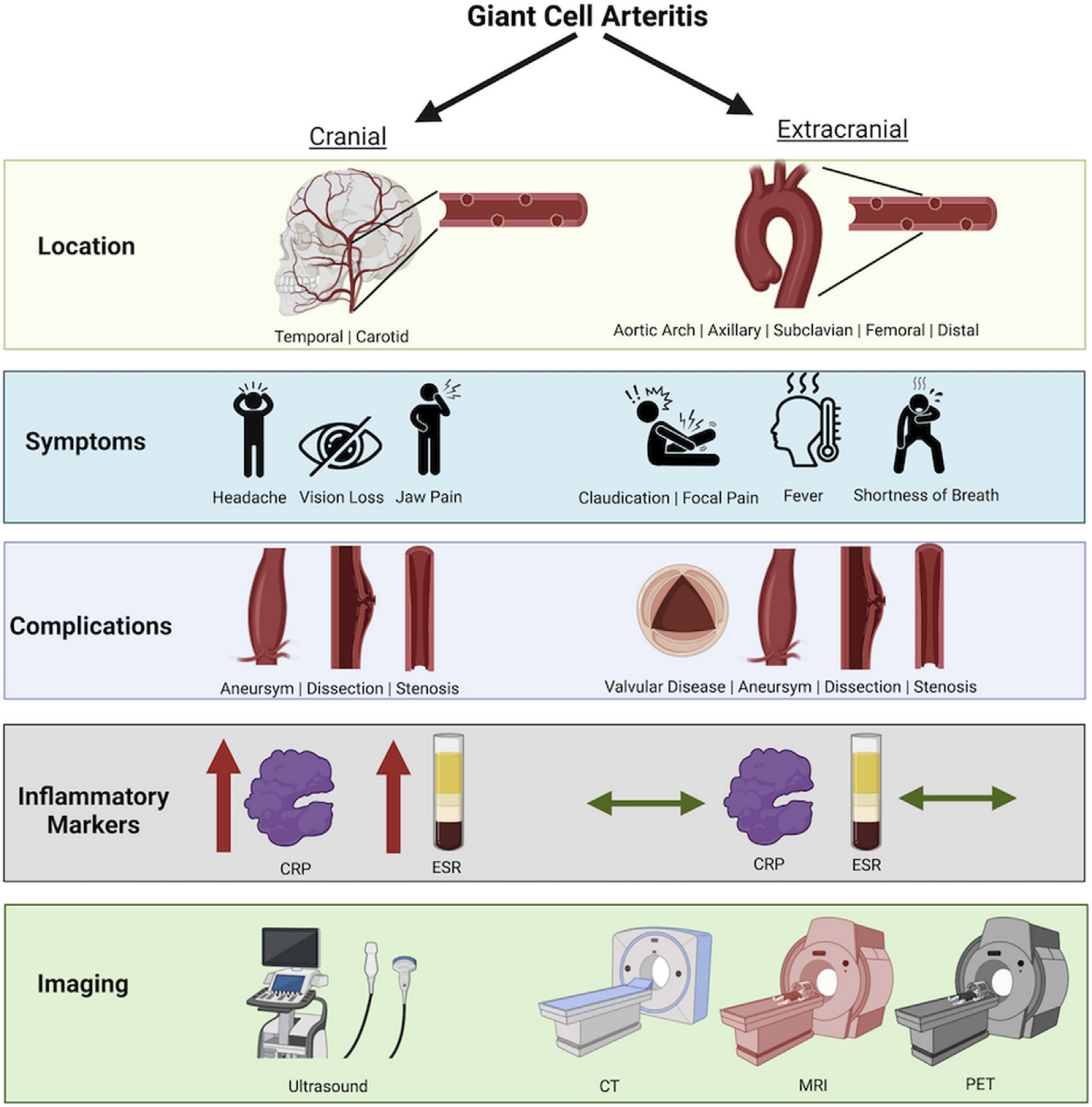
Differences Between Cranial and Extracranial Giant Cell Arteritis Differences between cranial and extracranial giant cell arteritis in anatomical locations, symptoms, complications, laboratory findings and imaging modalities. CRP= C-reactive protein; CT = computed tomography; ESR = erythrocyte sedimentation rate; MRI = magnetic resonance imaging; PET = positron emission tomography.

Figure 2 depicts the differences between cranial and extracranial GCA previously discussed.

### Management of GCA aortopathy

Once diagnosed, GCA aortopathy requires annual or biannual imaging surveillance (CT angiography or MRI) because of the risk of aneurysmal recurrence. Upon initial diagnosis, weight-based intravenous or oral steroids are used. Subsequent therapy involves glucocorticoid and nonglucocorticoid immunosuppressive agents such as biologic agents (tocilizumab) or oral immunosuppressants (methotrexate) as given by the 2021 American College of Rheumatology/Vasculitis Foundation guidelines for GCA and Takayasu arteritis.^10^

## Follow-up

The patient was administered weight-based steroids for 5 days and underwent transition to oral prednisone taper. He remained asymptomatic at discharge and received outpatient follow-up care with the rheumatology and cardiothoracic surgery services for nonglucocorticoid immunosuppressive therapy initiation and imaging surveillance.

## Conclusions

We discuss a unique case of asymptomatic extracranial giant cell aortopathy incidentally discovered during a mitral valve repair. Extracranial GCA remains unrecognized in clinical practice because of its asymptomatic or nonspecific clinical presentation and discordant inflammatory markers. Advanced imaging with CT or MRI angiography or PET-FDG is needed in diagnosing nonspecific or asymptomatic aortopathy because of the technical limitations of ultrasonography and noncontrast CT.

## Funding Support and Author Disclosures

Dr Khan and Ms Newstrom are Burroughs Wellcome Fund Scholars in the Texas A&M University Academy of Physician Scientists, supported in part by a BWF Physician Scientist Institutional Award. All other authors have reported that they have no financial relationships relevant to the contents of this paper to disclose.
